# Spectral CT in patients with acute thoracoabdominal bleeding—a safe technique to improve diagnostic confidence and reduce dose?

**DOI:** 10.1097/MD.0000000000016101

**Published:** 2019-06-21

**Authors:** Johannes Kahn, Uli Fehrenbach, Georg Böning, Felix Feldhaus, Martin Maurer, Diane Renz, Florian Streitparth

**Affiliations:** a Department of Radiology, Charité, Berlin; b Department of Diagnostic, Interventional and Pediatric Radiology, Inselspital, University of Bern, Bern; c Department of Radiology, University of Jena, Jena; d Department of Radiology, Ludwig-Maximilians-University, München, Germany.

**Keywords:** acute bleeding, dose reduction, multi-energy CT, practical implications, virtual native imaging

## Abstract

Computed tomography (CT) protocols for the detection of bleeding sources often include unenhanced CT series to distinguish contrast agent extravasation from calcification. This study evaluates whether virtual non-contrast images (VNC) can safely replace real non-contrast images (RNC) in the search for acute thoracoabdominal bleeding and whether monoenergetic imaging can improve the detection of the bleeding source.

The 32 patients with active bleeding in spectral CT angiography (SCT) were retrospectively analyzed. RNC and SCT series were acquired including VNC and monoenergetic images at 40, 70, and 140 keV. CT numbers were measured in regions of interest (ROIs) in different organs and in the bleeding jet for quantitative image analysis (contrast-to-noise ratios [CNR] and signal-to-noise ratio [SNR]). Additionally, 2 radiologists rated detectability of the bleeding source in the different CT series. Wilcoxon rank test for related samples was used.

VNC series suppressed iodine sufficiently but not completely (CT number of aorta: RNC: 33.3±12.3, VNC: 44.8 ± 9.5, *P* = .01; bleeding jet: RNC: 43.1 ± 16.9, VNC: 56.3 ± 16.7, *P* = .02). VNC showed significantly higher signal-to-noise ratios than RNC for all regions investigated. Contrast-to-noise ratios in the bleeding jet were significantly higher in 40 keV images than in standard 140 keV images. The 40 keV images were also assigned the best subjective ratings for bleeding source detection.

VNC can safely replace RNC in a CT protocol used to search for bleeding sources, thereby reducing radiation exposure by 30%. Low-keV series may enhance diagnostic confidence in the detection of bleeding sources.

## 1 Introduction

Computed tomography (CT) is widely used in patients with clinically suspected thoracoabdominal bleeding to identify the origin of bleeding and guide the treatment strategy. Because of its speed and wide availability, CT is the first-line imaging modality in emergency settings.^[1–4]^ It is generally recommended that the standard CT protocol for identifying bleedings should include an unenhanced series to distinguish contrast agent (CA) extravasation from calcifications or foreign bodies.^[5]^ After CA administration, arterial and venous phases are obtained to localize the bleeding source and assess its severity. With rising concern about carcinogenesis and other harmful consequences of medical radiation exposure,^[6,7]^ reducing dose has become an important objective for both radiologists and manufacturers. Virtual non-contrast (VNC) images could contribute to dose reduction by making real non-contrast (RNC) images obsolete. In our study, VNC images derive from spectral CT scans (Gemstone Spectral Imaging [GSI]), which are acquired by rapid switching between high and low tube voltages. This technical solution enables precise registration of datasets to generate material decomposition images (MDI) and virtual monochromatic spectral images (VMS), which can be used to subtract iodine from selected voxels and thus create VNC images. The clinical benefit of VNC images has been demonstrated in phantom studies^[8]^ as well as for different body regions and diseases including the liver,^[9]^ adrenals,^[10]^ kidneys,^[11,12]^ stomach tumors,^[13]^ and gastrointestinal bleeding.^[14,15]^ Moreover, spectral CT angiography (SCT) monochromatic series improve contrast-to-noise ratio (CNR), improving diagnostic accuracy in the search for metastases or the detection of renal lesions or by suppressing metal artifacts.^[16,17]^ The aim of this study was to evaluate SCT in 2 respects:

1. May VNC images safely replace RNC images in the detection of bleeding sources and
2. Do monochromatic low keV images improve the detection of bleeding sources?

## 2 Materials and methods

### 2.1 Patient characteristics and study design

From April 2016 until July 2018, a total of 198 patients with clinically suspected acute thoracoabdominal bleeding underwent SCT on a CT scanner with ultra-rapid kVp switching. 32 of these patients showed active arterial bleeding and met the following criteria, thus were included in this retrospective analysis:

(1) active thoracoabdominal visceral or soft tissue bleeding with visible CA extravasation jet in SCT;
(2) complete SCT protocol including RNC series, spectral mode arterial phase, standard mode venous phase, and reconstructed VNC series; and
(3) patient age >18 years.

Exclusion criteria were:

(1) absence of active bleeding (no CA extravasation jet);
(2) incomplete SCT protocol and
(3) patients being underage. The study was approved by the institutional ethics board.

### 2.2 CT technique

SCT multiphase scans were acquired on a 128-multislice CT scanner (Revolution HD, GE Healthcare, Milwaukee, WI) with ultra-rapid kVp switching (GSI). After acquisition of a posterior-anterior scout, an RNC series was acquired. Following administration of CA at a dose of 1.5 mL/kg body weight at a flow rate of 4 mL/s (max 120 mL, Xenetix 350, Guerbet, Villepinte, France), a spectral mode arterial phase (30 sec after injection) and a normal mode venous phase (120 sec after injection) were acquired. CT parameters (pitch, collimation, rotation time, slice thickness, iterative reconstruction level) were kept constant for acquisition of all series except for automated dose modulation in spectral CT mode (spectral CT does not feature this approach).

Spectral CT is an emerging multi-energy technique that allows for generation of different datasets including the above-mentioned MDI and VMS images by using projection-based reconstruction and rapid tube voltage switching between 80 and 140 kVp.^[18,19]^ In our study, we generated a virtual iodine-suppressed series by computing VNC images from VMS images acquired at 70 keV. Additionally, monochromatic images at 3 different energetic levels (40 keV, 70 keV, and 140 keV) were reconstructed.

### 2.3 Quantitative image analysis

All analyses were performed on a dedicated workstation (Advantage Workstation, GE Healthcare Milwaukee, WI). Circular regions of interest (ROIs) with an approximate size of 1 cm^2^ were placed in different organ regions (liver, pancreas, spleen, kidney, portal vein, aorta, subcutaneous abdominal fat, paraspinal muscle, air outside the body, and the CA extravasation jet) in spectral mode arterial phase images and then cloned to all other series.

Signal-to-noise ratio (SNR) and CNR were calculated. SNR was determined as the CT number of the specific body region divided by the standard deviation of noise: 
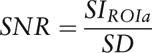


CNR was defined as the difference between any 2 tissues divided by the standard deviation of background noise: 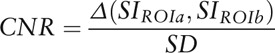


### 2.4 Qualitative Image analysis

Detectability of the bleeding source was rated by 2 experienced radiologists in consensus on the different CT series using a 5-point scale (with 5 indicating the best detectability).

### 2.5 VNC reconstruction time

VNC images are created semiautomatically. Once the reconstruction parameters for computation of the VNC series have been stored on the workstation, the VNC are computed in 3 steps and transferred to the PACS. The median time it took from selecting the patient on the workstation until images were available in the PACS was calculated.

### 2.6 Radiation dose

Radiation doses were estimated using the dose-length product (DLP) considering the individual scan length and the volume computed tomography dose index (CTDIvol).

### 2.7 Statistical analysis

All statistical analyses were performed using SPSS 23.0, (SPSS Inc., New York, NY). Values of *P* <.05 were considered statistically significant. Normal distribution of data was analyzed with the Shapiro–Wilk test (S–W test). Based on the results of the S–W test, a paired *t* test was performed for SNR and the Wilcoxon signed-rank test for applied doses, CNRs, and subjective image quality scores.

## 3 Results

### 3.1 Patient characteristics

A total of 198 patients with clinically suspected bleeding were examined using a dedicated SCT protocol. 32 of them (16.2%) showed active thoraco-abdominal or soft tissue bleeding and were included in our study. The patients had a mean age of 65.4 ± 14.1 years, and 23 of them were male. Data on bleeding sites are summarized in Table 1.

**Table 1:**
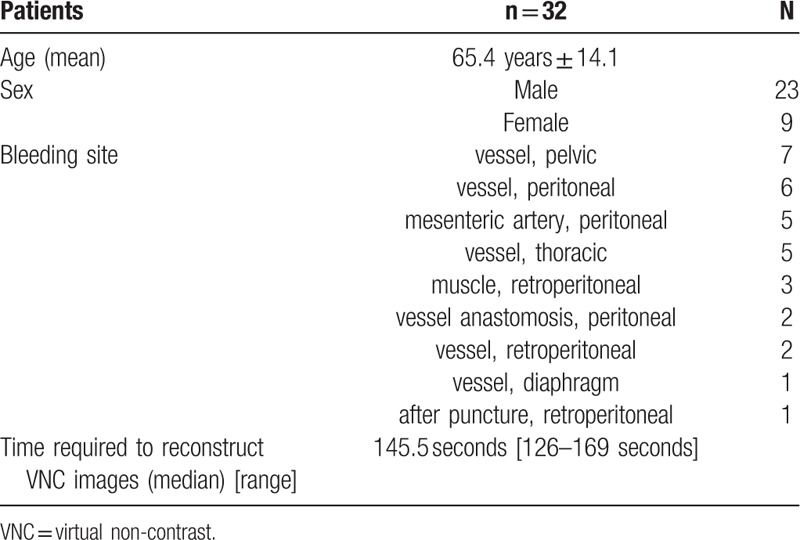
Patient characteristics, bleeding sites, and time to reconstruct VNC images.

### 3.2 Image quality and VNC reconstruction time

Except for liver parenchyma, all organ regions investigated had significantly higher mean CT numbers in VNC images than in RNC images. The VNC series showed sufficient but incomplete iodine suppression. The mean CT number in the aorta was 33.3 ± 12.3 in RNC images and 44.8 ± 9.5 in VNC images (*P* = .01); in the bleeding jet, the CT number was 43.1 ± 16.9 in RNC images and 56.3 ± 16.7 in VNC images (*P* = .02). Attenuation measurements showed increased levels in all organ regions at lower energies; monochromatic images at 40 keV showed significantly higher mean CT numbers in all organ regions compared to 140 keV images and partly compared to 70 keV images (see Table 2).

**Table 2:**
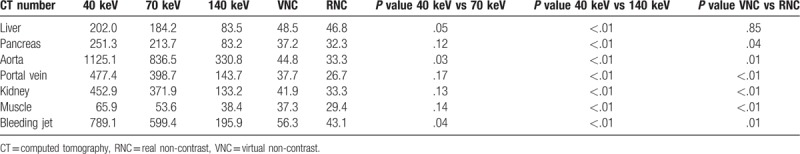
Results of quantitative image analysis: CT numbers (Hounsfield units) for 40, 70, and 140 keV, VNC, and RNC images.

SNRs were significantly higher in all organ regions in VNC images compared to RNC images as well as in most organ regions in 40 keV images compared to 140 keV images (see Table 3). SNRs between 40 and 70 keV did not differ significantly.

**Table 3:**
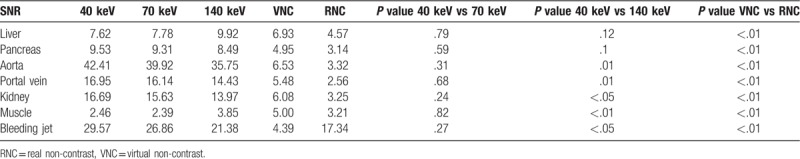
Quantitative image analysis: signal-to-noise ratios (SNRs) for the organ regions investigated.

CNRs did not differ significantly between VNC and RNC images except for the measurement in the bleeding jet, whereas 40 keV images showed significantly higher CNR values than 140 keV images but not than 70 keV images (see Table 4).

**Table 4:**
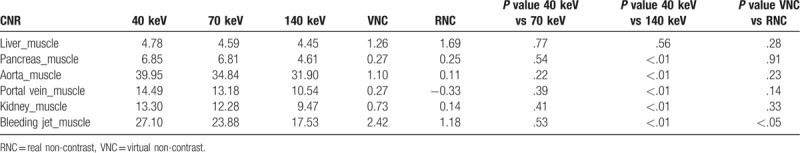
Quantitative image analysis: contrast-to-noise ratios (CNRs) relative to muscle tissue for the organ regions investigated.

There was good overall subjective image quality in 40, 70, and 140 keV images. We found an expected increase in contrast and image noise at low energy levels. Furthermore, diagnostic confidence for detection of the bleeding jet was significantly higher at lower energies, confirming the objective measurement that showed increased SNRs due to a relatively higher increase in useful signal compared to the increase in noise (see Table 5).

**Table 5:**
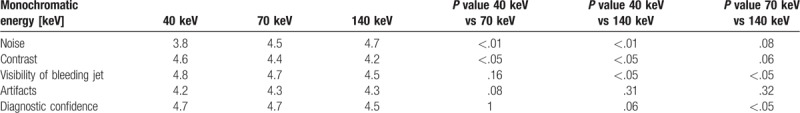
Qualitative image quality ratings using a Likert scale from 1 (poorest quality) to 5 (best quality).

The median time that was necessary to reconstruct VNC images was 145.5 seconds (see Table 1).

### 3.3 Radiation dose

**Figure 1:**
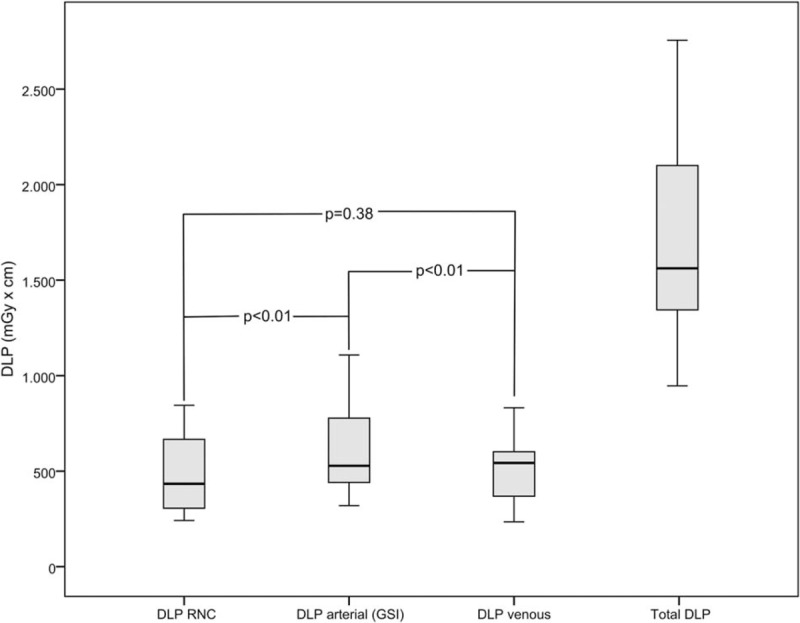
Boxplot of radiation dose exposures of the different contrast agent series constituting the protocol used in the study.

Mean total DLP was 1648 mGy∗cm (±487) with 492 mGy∗cm (±165) in the RNC series, 645 mGy∗cm (±207) in the spectral arterial series, and 517 mGy∗cm (±148) in the standard venous series. The DLP between RNC and arterial SCT series (*P* <.01) as well as between arterial SCT and venous series (*P* <.01) differed significantly. DLP between RNC and venous series did not show a significant difference (*P* = .38) (see Fig. 1). Correspondingly, RNC series accounted for 30% of the applied dose.

## 4 Discussion

Our results suggest that spectral CT imaging can improve the detection of thoracoabdominal bleeding sites in low keV monochromatic datasets. Furthermore, VNC series allow safe differentiation of calcifications or foreign bodies from CA extravasation making RNC series obsolete, thus reducing the radiation dose by approximately 30%.

We observed a significant increase in CNR and in SNR for 40 keV images compared to 140 keV images and a nonsignificant increase compared to 70 keV images, which correspond to standard 120 kV images in non-SCT scans. The higher scores for low keV datasets indicate that the increase in signal through greater iodine attenuation is relatively higher than the simultaneous increase in image noise. These objective measurements were confirmed by the radiologists’ subjective image quality ratings for overall diagnostic confidence in detecting the bleeding source, which was better for low keV images.

The observed increase in SNR in the VNC series compared to the RNC series may be explained by the higher attenuation of the measured organ regions in VNC images. At the same time, the standard deviation of background noise was lower in VNC images than in RNC images. Hence, the elevated SNR is the result of both the incomplete suppression of iodine and the reduced background noise in VNC images. Interestingly, only liver parenchyma did not show a significantly higher CT number in VNC images compared to RNC images. A possible explanation could be the fat content of the organ that may counterbalance the expected incomplete iodine suppression in VNC images and therefore may equalize the difference between VNC and RNC images of the liver.

**Figure 2:**
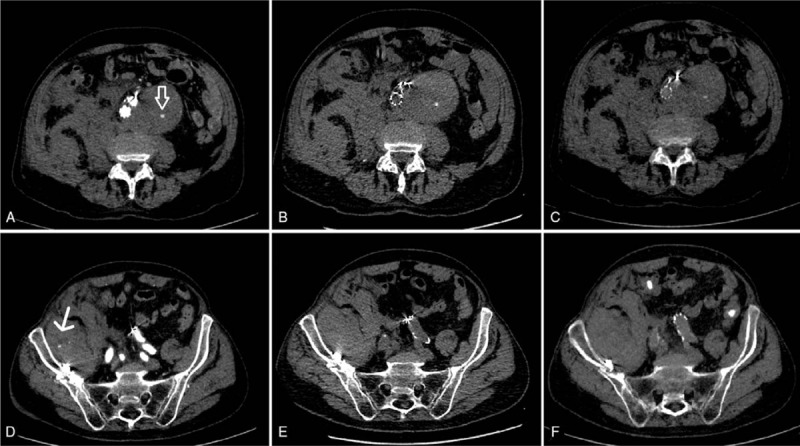
VNC series. 82-year-old male patient who presented to the ER with acute abdominal pain and a drop in Hb from 11.4 to 6.7 g/dL. CT shows a hyperdense dot in an aortic aneurysm (A—arrow) in the arterial contrast phase following EVAR. Both the real unenhanced (B) and virtual unenhanced phase (C) also show the hyperdense dot sign, consistent with calcification within the aneurysm. In this case, it would not have been possible to rule out active bleeding without an unenhanced CT phase. The same patient additionally shows a pelvic hematoma with a hyperdense dot in the arterial contrast phase (D—arrow). Both real unenhanced (E) and virtual unenhanced (F) scans fail to show a hyperdense dot sign, confirming active contrast agent extravasation consistent with active bleeding. CT = computed tomography, EVAR = endovascular aortic repair, VNC = virtual non-contrast.

**Figure 3:**
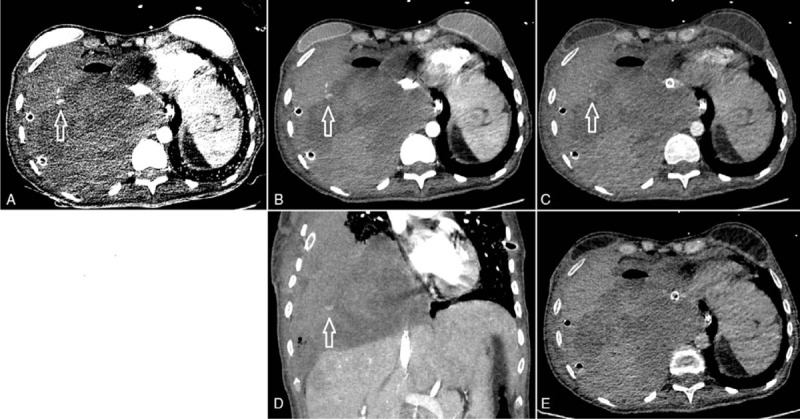
Arterial bleeding from an intercostal artery (arrows) with consecutive hemothorax. A: Monochromatic 40 keV image: high contrast-to-noise ratio with good demarcation of the arterial bleeding jet, improving identification of the arterial bleeding source. Note the high noise. SNR: 28, CNR: 21.4. B: (Standard) monochromatic 70 keV image (corresponding to monoenergy 120 kV image): good contrast-to-noise ratio. SNR: 25.4, CNR 19.3. C: Monochromatic 140 keV image: poor contrast of bleeding jet. SNR: 18.6, CNR 16.7. D: Coronal reconstruction of 70 keV image showing the bleeding jet. E: Virtual non-contrast image with no enhancement in the region of interest, consistent with contrast agent extravasation. CNR: 4.1, SNR: 2.6. All images are shown with the same windowing. Subsequent arteriography with occlusion of the fifth and sixth right intercostal arteries stopped the bleeding. CNR = contrast-to-noise ratio, SNR = signal-to-noise ratio.

Higher CT numbers for VNC images compared to RNC images have also been reported by Chai et al,^[13]^ who evaluated the feasibility of spectral CT imaging in gastric tumors. Our results are also in line with the findings of Yamada et al, who found less image noise in VNC images compared to real non-contrast images in cardiac patients.^[20]^ While complete iodine suppression is desirable, incomplete suppression as in our study does not degrade diagnostic accuracy. CA extravasation could be reliably differentiated from calcification in all cases analyzed in our study (see Fig. 2). Only a few studies investigated the detection of intestinal bleeding using dual-energy CT, including a study by Sun et al,^[14]^ who compared VNC and RNC images. Liu et al^[15]^ compared monochromatic versus polychromatic images to identify intestinal bleeding. Both studies found an improved detection of intraluminal bleeding sites using multi-energy CT. To the best of our knowledge, no study has been published on the evaluation of parenchymal bleeding sites in SCT that compares and combines the advantages of VNC and monochromatic imaging (see Fig. 3).

Radiation exposure has become an important concern in medical CT today. Replacing RNC images by VNC images may save approximately 30% of dose, corresponding to the contribution of RNC to the total dose of the triphasic protocol used in our study. We found spectral CT to have a slightly higher dose than standard CT series acquired after CA administration, but this effect is negligible compared with the dose reduction resulting from reducing the protocol to only 2 phases as an outcome of this study.

Another important factor is the implementation of new imaging techniques in daily clinical routine. With rising workloads for radiologists,^[21,22]^ it is highly desirable not to complicate image reading even further, which is especially important in emergency situations such as an acute bleeding. Once properly implemented, reconstruction of VNC images works semiautomatically based on a PACS-integrated solution. It is a simple “3-click” process that took approximately 2.5 minutes of the image postprocessing time. Overall, with omission of the RNC series, it does not take much longer until the complete image data are available.

Our study has some limitations. First, we used a retrospective study design with a relatively small number of patients due to the inclusion criteria. Second, our results are only valid for the CT scanner used in the study, which was the same for all examinations to ensure comparability of the patients.

In conclusion, VNC imaging can safely replace RNC imaging in a dedicated spectral CT protocol used to search for bleeding sources and can reduce the radiation dose by approximately 30%. Low keV series may improve diagnostic confidence in the detection of bleeding sites as a result of higher CT numbers, SNR, CNR as well as enhanced contrast and better visibility of the bleeding jet.

## Author contributions

**Conceptualization:** Johannes Kahn, Uli Fehrenbach, Georg Böning, Felix Feldhaus, Florian Streitparth.

**Formal analysis:** Johannes Kahn, Uli Fehrenbach, Florian Streitparth.

**Funding acquisition:** Florian Streitparth.

**Investigation:** Johannes Kahn, Uli Fehrenbach, Florian Streitparth.

**Methodology:** Johannes Kahn, Uli Fehrenbach, Florian Streitparth.

**Project administration:** Martin Maurer, Diane Renz, Florian Streitparth.

**Supervision:** Johannes Kahn, Florian Streitparth.

**Validation:** Uli Fehrenbach, Georg Böning, Felix Feldhaus.

**Writing – original draft:** Johannes Kahn, Uli Fehrenbach, Florian Streitparth.
